# Perspective on the 65-Year Anniversary of the Discovery of Cytomegalovirus

**DOI:** 10.3390/v17010080

**Published:** 2025-01-09

**Authors:** Edward S. Mocarski

**Affiliations:** 1 Emeritus, Department of Microbiology & Immunology, Stanford Medical School, Stanford University, Stanford, CA 94305, USA; mocarski@stanford.edu; 2 Emeritus, Department of Microbiology & Immunology, Emory Medical School, Emory Vaccine Center, Emory University, Atlanta, GA 30322, USA

This volume presents research and reviews bringing forward new insights into cytomegalovirus (CMV) pathogenesis and biology; CMV is a herpesvirus that has long been recognized as being medically significant. Human CMV (HCMV) causes serious disease following transplacental transmission during pregnancy, with consequent sensorineural damage to the newborn as well as in immunocompromised host settings such as following tissue or organ transplantation [1,2]. Infection by HCMV is widespread in humans but shows strict species specificity. It is impressive how much we have learned in the decades since the isolation of this virus from newborns with congenital infection [3,4] and its affirmation as a widespread infection with ready isolation from children and adults [5,6]. The first isolation and propagation of CMV (from laboratory mice) in cultured cells [7] revealed murine cytomegalovirus (MCMV), which, along with cytomegaloviruses of macaques, guinea pigs, and rats, became important surrogate models to study the biology of these species-restricted pathogens. Initially called salivary gland virus, CMV pathology had been recognized almost a century ago with observations on congenital disease in humans [8,9]. Similar cytopathology [10] and biology was shown in mice, the history of which was carefully assembled for the 60th anniversary celebration of cytomegalovirus isolation by Margaret Smith [11]. Professor Smith continued her research on virology until her retirement in 1964, imposed by an inability to convince newly established National Institutes of Health study sections that animal infections could be instructive regarding human viral disease [12]—a problem that continues to this day.

Here, research reports bring both clinical insights into HCMV disease as well as updates on the use of MCMV to gain insights that are difficult to approach with the human virus due to species specificity. Liberati et al. [13] have summarized public health awareness and interventions to reduce the burden of HCMV congenital infection and disease in a systematic review of the literature dealing with primary attention to awareness and screening through the end of 2023. The authors project that there is still inadequate attention paid to hygiene measures to prevent primary infection (or re-infection/reactivation) during pregnancy. Neonatal screening for congenital infection occurs in many locations around the world but does not follow an internationally accepted standard. Authors argue that larger studies pursuing screening and therapeutic intervention with valganciclovir are needed. The efficacy of past and ongoing vaccine clinical trials are summarized. Toriyabe et al. described a case study on a congenitally infected fetus [14], reporting a correlation between the more striking pathology and immunohistopathology and high CMV DNA levels in the thyroid gland, lungs, liver, and kidney, as well as lower immunohistopathology and CMV DNA levels in the cerebrum, thymus, heart, spleen, pancreas, and adrenal glands. This study should inspire greater awareness and further investigation into this important postmortem diagnostic issue.

Kobayashi and Hashida overview HCMV ocular disease [15], which runs a spectrum of virus-induced and immune response-induced sight-threatening syndromes (retinitis, corneal endotheliitis, and iridocyclitis, as well as uveitis) rising from congenital infection, as well as in both immunocompromised and, less frequently, immunocompetent adults.

Yeh et al. overview HCMV gastrointestinal disease [16], which is the most common invasive diseases caused by this virus at any anatomical location from the esophagus to the colon, primarily in individuals with other underlying serious illnesses or allogeneic transplants. The authors carried out a systemic review of the literature through mid-2022, finding that colon disease is most frequently described, suggesting that the disease is more common than widely perceived, particularly in high-risk patient populations. Yeh et al. [17] retrospectively evaluated over 400 patients diagnosed with HCMV gastrointestinal disease to assess susceptibility to disease in patients who are not traditionally viewed as immunocompromised; they reported that older age and gastrointestinal bleeding are important factors in both immunocompetent and immunocompromised patients, with an age >70 rather than >55 adding risk to the former group. In addition, immunocompetent patients did not benefit from 14 days or more of a treatment that is known to benefit immunocompromised patients, an observation that is confirmed here. Lin et al. [18] underscored the clinical significance of endoscopic biopsy and pathology in the accurate diagnosis of CMV colitis in patients following living donor liver transplantation.

Lee et al. [19] evaluated the risk of cardiovascular events and all-cause death among individuals with clinically apparent cardiovascular disease and found that the 20-year follow-up HCMV antibody levels correlate with all-cause death but not with cardiovascular outcomes in adults without pre-existing cardiovascular disease.

Chae et al. [20] evaluated the antiviral drug-resistant variants arising in 8% of 123 hematopoietic cell transplantation recipients who were unable to control human cytomegalovirus infection. Patients with variants who did not have their drug therapy modified did not control their infection and suffered a more severe graft-versus-host disease and reduced one-year survival.

Herbein and El Baba [21] examined the relationship of cytomegalovirus and cancer development, focusing on its contribution to polyploid giant cancer cell phenotypes. There is little question that this virus may be detected in patients with cancer, potentially due to the immunosuppressive nature of the cancer itself or the cancer therapy. The authors present their views on certain “high risk” strains of viruses they have studied that may drive polyploid giant cancer cells. Authors also describe transforming human cells in culture with the consistent retention of HCMV based on detecting immediate early gene expression.

A significant amount of research is carried out with human cell types in culture. Certain highly permissive cell lines, such as the retinal pigment epithelial cell line ARPE-19 have received a lot of experimental study. Golgonda et al. [22] raise concerns regarding investigations into the cellular transcriptome and behavior of subconfluent ARPE-19 cells. These cells are commonly employed to model the epithelial cell response to HCMV infection and revealed important features of HCMV attachment and entry. They report that RNAseq data show that APRE-19 cells resemble mesenchymal fibroblasts more closely than they do cells with “strong epithelial character” and that when other epithelial cells are infected with HCMV, they “exhibit an atypical infectious cycle and naturally restrict the production of cell-free progeny”, which is distinctly different from the behavior of APRE-19 cells. The work raises questions as to what cell type subconfluent APRE-19 cells really represent.

Zeng et al. [23] review the diversity of protein-coding messenger (m)RNAs, as well as non-coding RNAs expressed from the HCMV 235 kilobase pair genome during active replication (so-called lytic gene expression) as well as during latency. Recent studies have indicated that this virus does not show qualitative differences between the two types of infection but rather quantitative differences, with a much lower abundance of single RNA patterns characterizing the natural settings of persistence and latency [24,25]. Four types of RNA are expressed by this virus, i.e., linear mRNA and circular (c)RNA that encode proteins, together with long non-coding (lnc)RNA and micro (mi)RNA. The literature describing the activities of HCMV genes has grown tremendously, and multiple interpretations are possible from data presented with laboratory-propagated strains of the human virus. An extensive table provides information relating to the complex viral transcriptome, complementing and providing insights like those published in the last edition of *Fields Virology* [2].

HCMV encodes many gene products that manipulate various aspects of the immune response during infection. Wang et al. [26] investigate the immunomodulatory impact of HCMV UL23 in relation to the antiviral effect of IFNγ, and suggest that this occurs through the downregulation of the expression of APOL1 (Apolipoprotein-L1), CMPK2 (Cytidine/uridine monophosphate kinase 2), and LGALS9 (Galectin-9), which are all host proteins that may impede viral replication.

Lujan et al. [27] present a comprehensive current understanding and antiviral potential of the complement system on herpesviruses, as well as the ways in which viruses subvert the activation of the complement. While there is evidence of the viral subversion of the complement system, the understanding is notably underdeveloped, particularly for cytomegaloviruses; therefore, the authors point out directions for future study and the potential implications for understanding immunity and vaccine potential.

Animal models of CMV infection and disease have consistently given insights into its pathogenesis. Liu et al. [28] have summarized hearing impact findings from animal models studying surrogate cytomegaloviruses (murine and guinea pig), as well as other viruses, suggesting that inflammatory pathways may be important to consider in the pathogenesis of HCMV congenital disease. Chen et al. [29] have revealed that mesenchymal stem cell-derived exosomes afford therapeutic benefit in a mouse model of CMV pneumonitis, opening the way for further investigations into this adjunct approach to antiviral therapy. Smith et al. summarize efforts to make use of RNase P ribozymes in antiviral therapy [30]. The experimental fusion of an external guide sequence to an M1 ribozyme provides sequence targeting and subsequent optimization through various selection protocols to strengthen ribozyme–substrate affinity. RNase P has been directed at various human and MCMV gene targets with therapeutic success in cell culture and the mouse model. Efforts to further optimize specificity and activity are foreseen in advance of clinical therapeutic intervention using this strategy

Karner et al. [31] employ a mouse model of CMV infection to survey selected cytokine, chemokine, and growth factor levels in the liver, lungs, spleen, and brain after inoculating newborn mice intraperitoneally with a low dose (400 PFU) of MCMV on postnatal day 0 or 1. Because the mouse virus does not cross the placenta, these conditions are employed to model HCMV congenital infection. Notably, the work affirms observations on elevated proinflammatory cytokines and revealed that CC chemokines were broadly upregulated in the brain at two weeks post-inoculation as the mice developed the disease.

Finally, Zeng et al. [32] review the current body of research on CMV-based vectors, including the large body of work that has shown that rhesus CMV bacmid-derived vectors expressing simian immunodeficiency virus antigens protect against challenge and induce potent T cell responses against vectored antigens in rhesus macaques, despite their existing immunity to cytomegalovirus. This work and other more limited studies of MCMV-based vectors (as well as others) may inform the design of HCMV vaccine vectors. Fascinating experimental uses of MCMV bacmid-based vectors encoding either telomerase reverse transcriptase or follistatin were shown to be capable of extending the lifespan of laboratory mice [33]. Live attenuated HCMV has been tested for vaccine potential but its use as a vaccine or a gene therapy vector has not been reported.

The continuing worldwide impact of HCMV infection, despite available antiviral drugs such as valganciclovir, reinforces the need for the continued investigation of the molecular virology of this virus and its animal homologs. Decades of effort towards a universal HCMV vaccine capable of preventing congenital disease have yet to deliver. Control over this unceasing infectious threat will require continued public health investment and awareness in partnership with objective scientific research at both basic and clinical levels.
